# Buffalo City Dispensary

**Published:** 1874-02

**Authors:** 


### Buffalo City Dispensary.

We are gratified to see that the old Buffalo City Dispensary is again in active operation. This institution has been more truly humane in its scope and influence than any other with which we are acquainted in the city. The poor not under the care of the poor department, have, through its provisions, been able, when sick, to obtain satisfactory medical advice, and carefully prepared, pure medicine.

When the new "Free Dispensary," so called, entered the field as a sort of rival for dispensing charity, the long established and most humanely conducted Buffalo City Dispensary for a time closed its doors, hoping, as we suppose, there would be no further need of its benefits. Time seems to prove that its field of usefulness was in no way lessened and its board of officers have again opened its doors to the destitute sick.

The following medical and surgical staff was appointed for the ensuing year:

J. F. Miner, M. D., Consulting Surgeon.

T. M. Johnson, M. D., " "

J. P. White, M. D., Consulting Physician.

T. F. Rochester, M. D., " "

Physicians.—Wm. Ring, M. D., J. B. Samo, M. D., M. G. Potter, M. D., J. Hauenstein, M. D., L. Krombein, M. D.

Apothecaries.—W. H. Peabody, C. M. Lyman and J. P. Deihl.

Officers.—F. P. Wood, Esq., President.

Geo. A. Moore, Esq., Vice-President.

S. N. Callender, Esq., Treasurer.

Isaac D. White, Secretary.

